# Ropeginterferon alfa-2b as a promising alternative to conventional interferon in CDA type 1: a case report of two siblings

**DOI:** 10.1007/s10238-025-01785-x

**Published:** 2025-07-10

**Authors:** Tyng-Wei Yang, Xavier Cheng-Hong Tsai, Yu-Hsuan Fu, Liang-In Lin, Hsin-An Hou

**Affiliations:** 1 Division of Hematology, Department of Internal Medicine, National Taiwan University Hospital, Taipei, Taiwan; 2 Department of Hemato‑Oncology, Cathay General Hospital, Taipei, Taiwan; 3 Department of Clinical Laboratory Sciences and Medical Biotechnology, National Taiwan University, Taipei, Taiwan; 4 Division of General Medicine, Department of Internal Medicine, National Taiwan University Hospital, Taipei, Taiwan

**Keywords:** CDA, Ropeginterferon alfa-2b, CDAN1

## Abstract

Congenital dyserythropoietic anemia type I (CDA-I) is a rare hereditary anemia caused by *CDAN1* or *C15orf41* mutations, with *CDAN1*-related cases responding to interferon-alpha (IFN-α) therapy. However, traditional IFN-α requires frequent injections and often causes flu-like symptoms, which can hinder long-term adherence. Here, we present the first documented use of ropeginterferon alfa-2b, a next-generation pegylated interferon, in two patients with CDA-I. Both patients, who were previously transfusion dependent, achieved transfusion independence and improved hemoglobin levels after transitioning from recombinant IFN-α to ropeginterferon alfa-2b with biweekly dosing. This treatment preserved efficacy while minimizing adverse effects and the injection burden. Our findings suggest that ropeginterferon alfa-2b may serve as a more tolerable and effective long-term treatment. Further prospective large-scale studies are needed to validate its broader clinical applicability.

## Introduction

Congenital dyserythropoietic anemia (CDA) is a diverse group of inherited disorders characterized by ineffective erythropoiesis. CDA is classified into five types (CDA-I, II, III, transcription factor-related CDAs, and CDA variants) [1], which were historically distinguished based on clinical presentation and erythroblast morphological changes. In CDA-I, for example, erythroblasts typically exhibit a “spongy heterochromatin” or “Swiss cheese” appearance when examined by scanning electron microscopy (SEM). Advancements in molecular genetics have led to the identification of pathogenic genes responsible for CDA. CDA-I is caused by biallelic mutations in either the *CDAN1* or *C15orf41* gene. To date, 56 causative mutations have been documented, with 51 occurring in the *CDAN1* gene and 5 in the *C15orf41* gene [2].

CDA treatment was limited to supportive care for many years until 1995, when Lavabre-Bertrand et al. unexpectedly reported that interferon-alpha (IFN-α) was effective in treating CDA-I in a 28-year-old patient with concomitant CDA-I and hepatitis C [3]. However, its requirement for thrice-weekly subcutaneous injections can significantly impact quality of life. Here, we describe two CDA-I patients who achieved transfusion independence and improved hemoglobin levels with ropeginterferon alfa-2b, a next-generation pegylated interferon requiring less frequent administration. This treatment may offer a practical and well-tolerated option for managing CDA-I.

## Case presentation

A 29-year-old woman presented to a hematology clinic with intermittent shortness of breath persisting for several years. Although she was diagnosed with alpha-thalassemia minor at birth, she remained asymptomatic throughout childhood. She had no skeletal or limb abnormalities, chest deformities, or short stature. Her first visit to a pediatric hematologist was at the age of 15, when microcytic anemia (hemoglobin 7.0 g/dL, MCV 71.7 fL) and indirect hyperbilirubinemia (total/direct bilirubin = 6.23/0.7 mg/dL) were documented (Table 1). At that time, genetic testing revealed a heterozygous alpha-globin gene deletion (-SEA), leading to a diagnosis of α-thalassemia. Her red blood cell morphology revealed only anisopoikilocytosis. Among her family members, including three siblings, only the proband and one elder sister exhibited significant hemolytic anemia, despite both the father and the same sister having α-thalassemia minor.

**Table 1:** Laboratory values of the patient

Test	Value			Reference range
	15-year-old	Before IFN-α treatment	1 year after IFN-α treatment	
Red blood cell count (M/µL)	3.11	3.06	4.64	3.7–4.96
Hemoglobin (g/dl)	7.0	7.1	10.7	10.2–13.2
Hematocrit (%)	22.3	23.9	36.4	32.4–41.2
Mean corpuscular volume (fL)	71.7	78.1	78.4	76.9–93.7
Platelet count (K/µL)	237	190	163	186–353
Reticulocyte (%)	1.07		1.35	0.9 ~ 1.94
White blood cell count (K/µL)	6.41	3.74	4.93	4.19–11.4
Blast (%)	0	0	0	
Promyelocyte (%)	0	0	0	
Myelocyte (%)	0	0	0	
Metamyelocyte (%)	0	0	0	
Band neutrophil (%)	0	0	0	0–5
Segmented neutrophil (%)	61.1	62.9	54.1	41.6–74.4
Eosinophil (%)	1.9	2.4	1.4	0.3–7.9
Basophil (%)	0.2	0.5	0.2	0.2–1.6
Monocyte (%)	2.3	2.9	3.7	3.3–8.9
Lymphocyte (%)	34.5	31.3	40.6	3.3–8.9
Normoblasts (/100WBC)	0	0	0	
Total bilirubin (mg/dL)	5.19	5.35	2.63	0.2–1.2
Direct bilirubin (mg/dL)	0.63	0.54	0.45	0–0.4
Alanine aminotransferase (U/liter)	7	12	19	< 31
Lactate dehydrogenase (U/liter)	439	248	173	230–460
Hb-EP(HbH)(%)	Negative			
Hb-HPLC(HbA2)(%)	2.2			< 3.4
Hb-HPLC(HbF)	3.1			< 1.9

Since the age of 20, she began requiring regular blood transfusions every 2–4 months to alleviate exercise intolerance and dizziness and was prescribed folic acid supplementation. However, because her anemia was significantly more severe than expected for thalassemia minor alone, comprehensive testing, including hemoglobin electrophoresis, the eosin-5'-maleimide (EMA) binding test, the osmotic fragility test, and fluorescently labeled AERolysin (FLAER) flow cytometry, yielded normal results.

To further investigate the underlying cause of her hemolytic anemia, whole-exome sequencing followed by Sanger sequencing was performed. This analysis revealed compound heterozygous mutations in the *CDAN1* gene: c.2140C > T (p.R714W), inherited from her mother, and c.2059C > T (p.R687C), inherited from her father. Her elder sister, who exhibited a similar clinical presentation, was also screened, with results revealing that she also carried the same heterozygous mutations. Based on these findings, the patient was diagnosed with α-thalassemia minor with CDA-I.

**Fig. 1:**
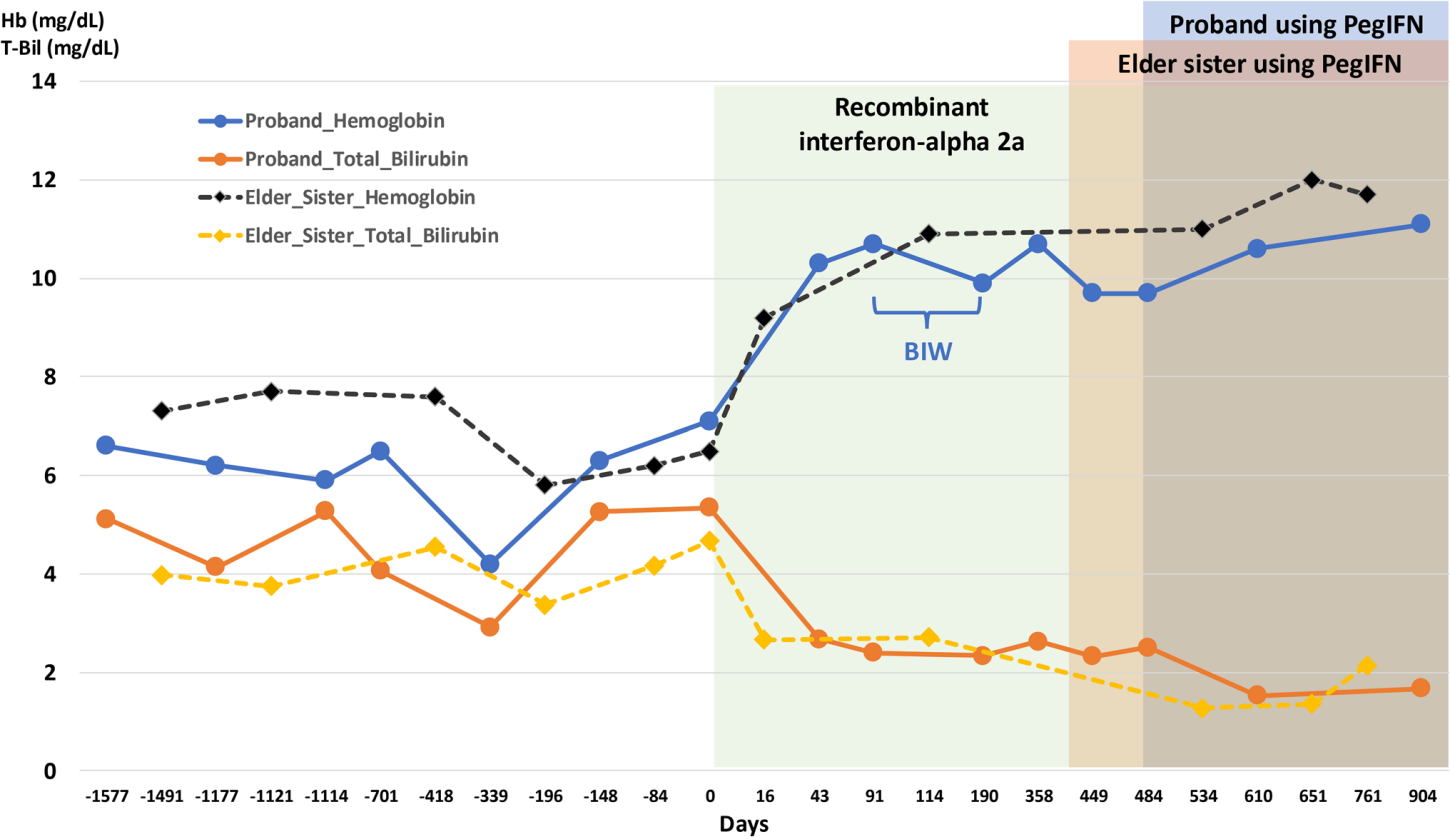
Hemoglobin and total bilirubin (T-Bil) levels over time in the proband and her elder sister with CDA-I, with Day 0 marking the start of IFN treatment. The proband’s hemoglobin (blue solid line, circular markers) and total bilirubin (orange solid line, circular markers) improved after treatment initiation. The elder sister’s hemoglobin (black dashed line, diamond markers) and total bilirubin (yellow dashed line, diamond markers) levels followed similar trends. The green shaded area represents recombinant interferon-alpha 2a therapy, during which the proband’s dosing was adjusted to twice weekly owing to intolerance. The blue- and brown-shaded areas indicate the transition to ropeginterferon alfa-2b for the proband and her sister, respectively. Both patients demonstrated a sustained increase in hemoglobin and a decrease in T-Bil under ropeginterferon alfa-2b therapy (colour figure online)

After confirming the diagnosis and having thorough discussions, we initiated treatment with recombinant interferon alfa-2a (Feronsure®) at a dose of 3 million IU three times per week. The patient demonstrated a significant response, with hemoglobin increasing from 7.1 g/dL to 10.3 g/dL and total bilirubin decreasing from 5.35 mg/dL to 2.68 mg/dL after six weeks of therapy (Fig. 1). However, frequent injections resulted in significant intolerance, including local injection site pain and fever, which adversely affected her quality of life. To mitigate these side effects, the Feronsure® dosage was tapered to two times per week, but this resulted in a decrease in hemoglobin levels. We sought an alternative to this long-term treatment which could last decades, as an approach to balance efficacy and tolerability. After 16 months on Feronsure®, we initiated treatment with ropeginterferon alfa-2b (Besremi®), which was administered biweekly, starting at a dose of 100 µg and gradually titrating up to 250 µg. The patient tolerated ropeginterferon alfa-2b well, with stable hemoglobin levels and no significant adverse effects. Notably, the same treatment approach was used for her older sister, who also showed a positive response and experienced a notable improvement in her quality of life.

## Discussion

The diagnosis of rare inherited anemias, such as CDA-I, has traditionally depended on bone marrow examination and erythroblast morphology assessment, often leading to delayed recognition or misdiagnosis [4, 5]. Advances in next-generation sequencing have transformed this process, enabling earlier and more precise diagnoses while reducing reliance on invasive procedures. As molecular analysis becomes more accessible, genetic testing is now the preferred first-line tool for diagnosing CDA [1, 4], but biochemical and morphological assessments still play a role in resolving cases with uncertain genetic variants.

CDA is a group of rare congenital hypoproliferative anemias. Among its subtypes, CDA-I, an autosomal recessive disease caused by either biallelic *CDAN1* or *C15orf41* mutations, accounts for only 4.4% of all CDA cases [1]. Notably, CDA-I is the only CDA subtype that responds to IFN-α therapy. [6] Although the underlying mechanism remains unclear, a study by Wickramasinghe et al. revealed that CDA-I patients exhibit reduced IFN-α production compared with both healthy controls and patients with other CDA subtypes, possibly explaining its therapeutic benefit. [7] Table 2 summarizes previous CDA treatment approaches and their corresponding responses. Among CDA-I patients, the response to IFN-α varied, but the key observation is that continuous injections were necessary to maintain hemoglobin levels. Since the first report of IFN-α as an effective treatment for CDA-I in 1995, [3] its use has been limited by the need for thrice-weekly or twice-weekly injections, which affects long-term adherence. Subsequent reports have documented similar therapeutic benefits in CDA-I patients, further supporting its role as a disease-specific treatment [8–11]. While both *CDAN1* and *C15orf41* mutations cause CDA-I, a study reported that only patients with *CDAN1* mutations respond well to IFN-α treatment, whereas those with *C15orf41* mutations may not [12]. This difference could explain why not all CDA-I patients respond equally to IFN-α therapy. The differential interferon responsiveness between patients with *CDAN1* and *C15orf41* mutations remains incompletely understood. *CDAN1* encodes Codanin-1, a protein implicated in chromatin assembly and nuclear envelope stability during erythropoiesis [13]. Dysregulation of Codanin-1 may render erythroid precursors more sensitive to the transcriptional and epigenetic modulation triggered by IFN-α. In contrast, *C15orf41* is predicted to encode an endonuclease with potential roles in DNA repair or the DNA replication stress response [12]. These pathways may be less amenable to modulation by interferon signaling. Functional studies exploring interferon-induced transcriptional changes in *CDAN1*- versus *C15orf41*-mutated cells are needed to substantiate this hypothesis. However, because of the limited number of cases and the low availability of genetic testing, it remains unclear whether this difference in response rates is conclusive. While traditional IFN-α effectively treats CDA-I, its frequent dosing regimen poses significant quality-of-life challenges. Ropeginterferon Alfa-2b (Besremi®), a next-generation pegylated interferon, offers a longer half-life, allowing for biweekly administration.

**Table 2:** A brief review of prior treatment approaches for CDA and their corresponding responses

References	Age	Sex	CDA type	Dx method	Tx agent	Dose	Hb (g/dL)		Addendum
							Before	After	
Lavabre-Bertrand et al.[9] (1995)	28	F	I	SEM	IFN-alpha 2a	3MU TIW × 24 wks	8.6	11.5	Responsive but requiring continuous treatment
Wickramasinghe et al.[11]	30	F	I	LM	IFN-alpha 2a	3 M IU TIW × 5 wks	8.2	12.1	Responsive
Shamseddine et al.[23]	31	M	I	LM	IFN-alpha	3 M IU TIW × 16 wks	8.4	12	Responsive. The patient stopped the drug spontaneously. Hb remained stable for another 2 months then gradually dropped to 8.5 g/dL at 14 months after treatment initiation
	64	F	I	LM	IFN-alpha	3 M IU TIW × 12 wks	8.8	11.0	Responsive, but the Hb level dropped to pretreatment level after discontinuing IFN-alpha for 7 months
Marwaha et al.[6]	2.5	F	I	LM	IFN-alpha 2b	4.2 M IU/m^2^ TIW x 13wks	8.5	4.0	No response on Hb, transfusion requirements, or the size of the spleen and liver
	7	M	I	LM	IFN-alpha 2b	3.9 M IU/m^2^ TIW × 15 wks 6.5 M IU/m^2^ TIW × 15 wks	8.0	9.0	
	11.5	M	I	LM	IFN-alpha 2b	2.6 M IU/m^2^ TIW × 9 wks 2.6 M IU/m^2^ QIW × 10 wks	9.0	8.0	
	5.5	M	II	LM, Ham’s test	IFN-alpha 2b	4.3 M IU/m^2^ TIW × 8 wks 4.3 M IU/m^2^ QIW × 4 wks	4.0	4.0	
	6 m	F	II	LM, Ham’s test	IFN-alpha 2b	5 M IU/m^2^ TIW × 6 wks 5 M IU/m^2^ QIW × 17 wks	8.5	5.5	
	8	M	II	LM, Ham’s test	IFN-alpha 2b	4.6 M IU/m^2^ TIW × 15 wks	10.0	4.0	
Agrigento et al.[8]	15	F	I	SEM	IFN-alpha 2b	4.5 M IU QW	8.5	9.44	Increased Hb level but hemolysis persisted *This patient is also a Sicilian Beta Thalassemia carrier
	50	F	I	SEM	IFN-alpha 2b	9 M IU QW	6.62	7.17	Partial response with prolonged blood transfusion interval (30 days to 49 days) *This patient is also a Sicilian Beta Thalassemia carrier
Rathe et al.[10]	11 m	M	I	*C15orf41* (p.Leu178Gln)	Peg-INF-alfa-2a	45mcg QW × 36 wks	9.8	10	Stable hemoglobin and ferritin level

Abbreviations: IFN = Interferon; SEM = Scanning electron microscope; LM = Light microscope; TIW = Thrice weekly; QIW = Four times weekly; Ham’s test = Acidified serum lysis test

Pegylated interferon alfa-2a has demonstrated comparable or superior efficacy and a similar safety profile to conventional IFN-α in various disease settings, including chronic hepatitis C [14]. Ropeginterferon alfa-2b demonstrated greater drug exposure with a comparable safety profile to pegylated IFN alfa-2a in a phase I trial [15]. In patients with polycythemia vera, ropeginterferon also achieved a faster therapeutic response and was associated with significantly fewer flu-like side effects than peginterferon alfa-2a [16]. Given its enhanced efficacy and improved tolerability, ropeginterferon alfa-2b represents a preferred option when the immunomodulatory properties of IFN-α are desired.

In addition to its extended half-life and improved tolerability, ropeginterferon alfa-2b has pleiotropic immunomodulatory effects that may contribute to its therapeutic effect on CDA-I. Interferon-α is known to induce a wide array of interferon-stimulated genes involved in hematopoietic regulation, antiviral defense, and apoptosis. In erythroid precursors, IFN-α signaling has been shown to suppress ineffective erythropoiesis, enhance terminal maturation, and potentially alter the epigenetic landscape via JAK-STAT activation and histone-modifying enzymes [17–19]. These effects may help shift erythroid precursors toward more functional maturation in CDA-I patients.

While ropeginterferon alfa-2b has demonstrated a favorable safety profile in myeloproliferative neoplasms, potential risks such as autoimmune manifestations (e.g., thyroiditis, lupus-like syndromes) [20] and abnormal liver function[21] have been reported and warrant close monitoring. In our patients, no significant immune-related adverse events or hepatotoxicity were observed during follow-up.

Notably, with respect to dosing considerations, the study by Miyachi et al. reported that systemic exposure to ropeginterferon alfa-2b in Asian individuals was approximately 1.7- to 2.0-fold greater than that in Caucasian subjects [22]. Although the incidence of adverse events did not significantly differ between the two populations, these pharmacokinetic differences suggest that a more conservative dose-escalation strategy may be appropriate for Asian patients.

Our report is the first to show that ropeginterferon alfa-2b remained effective in two patients with CDA-I but enhanced their quality of life by reducing the burden of injections. Both patients achieved optimal disease control with a biweekly dose of 250 µg, achieved normal hemoglobin levels and improved hyperbilirubinemia. Given the rarity of CDA-I, this study highlights the potential of pegylated interferon as a viable long-term treatment strategy. In summary, we present the first documented use of ropeginterferon alfa-2b in CDA-I patients, demonstrating its efficacy with less frequent dosing and better tolerability than traditional IFN-α. These cases underscore the role of genetic testing in CDA-I diagnosis and highlight pegylated interferon as a potential long-term treatment option. Further research is needed to validate its broader clinical utility.

## Data Availability

No datasets were generated or analysed during the current study.
